# Association of changes in commute mode with body mass index and visceral adiposity: a longitudinal study

**DOI:** 10.1186/s12966-019-0870-x

**Published:** 2019-11-06

**Authors:** Keisuke Kuwahara, Hisashi Noma, Tohru Nakagawa, Toru Honda, Shuichiro Yamamoto, Takeshi Hayashi, Tetsuya Mizoue

**Affiliations:** 1 Department of Epidemiology and Prevention, Center for Clinical Sciences, National Center for Global Health and Medicine, Tokyo, Japan; 2 Teikyo University Graduate School of Public Health, 2-11-1 Kaga, Itabashi-ku, Tokyo, 173-8605 Japan; 3 The Institute of Statistical Mathematics, Tokyo, Japan; 4 Hitachi Health Care Center, Hitachi, Ltd, Ibaraki, Japan

**Keywords:** Active commuting, Public transport use, Body weight control, Cohort studies, Asia

## Abstract

**Background:**

Prospective data are sparse for active commuting to work and body weight in Asia. We assessed the association of 5-year changes in commuting mode with body mass index (BMI) and the indicators of abdominal obesity in Japanese working adults.

**Methods:**

In this longitudinal study, we studied 29,758 participants (25,808 men and 3950 women) in Japan aged 30 to 64 years at baseline who underwent further health examination 5 years after the baseline examination. Changes in BMI were calculated from objectively measured body height and weight at baseline and follow-up examination. Visceral and subcutaneous fat areas and waist circumference measured by computed tomography scans were used as indicators for abdominal adiposity. Linear regression was applied to estimate the association of changes in commuting mode with the obesity outcomes.

**Results:**

Within the 5-year study period, adults who maintained inactive commuting gained weight, and compared with this group, adults who switched to inactive commuting had higher weight gain; conversely, adults who switched to active or public transportation commuting and especially those who maintained active or public transportation commuting experienced less weight gain. Subgroup analysis showed similar tendency across the different transitions of leisure-time exercise or occupational physical activity. For example, among adults who maintained no exercise (*n* = 16,087), the adjusted mean (95% confidence intervals) of the BMI change over 5 years in kg/m^2^ were 0.27 (0.24 to 0.30) for maintained inactive commuting group (reference), 0.34 (0.30 to 0.38) for switching to inactive commuting group (*P* = 0.046), 0.20 (0.18 to 0.22) for switching to active commuting or public transportation group (*P* = 0.001), and 0.09 (0.06 to 0.13) for maintained active commuting or public transportation group (*P* < 0.001). Maintained inactive commuting tended to be associated with larger gain in abdominal adiposity indicators.

**Conclusion:**

Switching from inactive commuting mode to more physically active commuting mode and maintaining active commuting can promote body weight control among working adults across different levels of occupational or leisure-time physical activity.

## Introduction

The prevalence of obesity has substantially increased worldwide over 30 years [1], imposing tremendous disease burden [2]. Obesity is particularly prevalent among young to middle-aged adults [3], suggesting that body weight management is critically important in this age group. For many adults, commuting to work is a routine part of daily life. However, car commuting, which is a physically inactive activity, has been the dominant mode of commuting in developed countries [4, 5] including Japan [6]. Moreover, the number of private car owners is growing rapidly in developing countries [7], raising numerous health and environmental concerns [7, 8]. Changing from physically inactive commuting to more active commuting (e.g., walking, cycling, or public transportation use) has been considered as a promising strategy against issues of not only obesity control but also traffic, green gas emission, air pollution, and noise [9–11], as also suggested by the World Health Organization [12, 13].

Accumulating evidence suggest that active commuting may help body weight control, whereas prolonged car use may not. However, several points should be further considered. First, most data were derived from cross-sectional studies [14–18], which are limited to infer causality. Second, although some investigated the prospective association of active commuting or car use with body mass index (BMI) [19–24] or obesity [25], two used the exposure measured only at one-time point (at baseline) [24, 25]. Additional data focusing on changes in commuting mode are warranted to establish solid evidence. Third, in five of the seven studies, BMI was estimated based on self-report [19, 21–24], which may not be accurate. Further investigations using objective measurement of BMI are needed to quantify the association between commuting and BMI more precisely. Fourth, these prospective data were obtained in Europe [19–21, 23, 25] or Australia [22, 24], and no data are available in Asian countries, where the average BMI level is lower than that in Western countries [26]. Given that the mean BMI levels and prevalence of obesity have been increasing in Asia [26], evidence to estimate the effects of active commuting to work on BMI is important in Asia to advance health policies for promoting active commuting. Lastly, no prospective data are available regarding the association between active commuting and visceral adiposity, which is a key component of obesity [27].

Herein, we report the 5-year changes in commuting mode to work with simultaneous changes in objectively measured obesity indicators including BMI, waist circumference, and visceral and subcutaneous fat measured via computed tomography (CT) scans among adults with majority of men. We also investigated the commuting-BMI associations according to the leisure-time or occupational physical activity transitions, which may have an impact on the magnitude of BMI change.

## Methods

### Study settings

The J-ECOH Study is an ongoing collaborative study to elucidate the risk factors for non-communicable diseases among workers in Japan from more than 10 large-scale companies. We analysed longitudinal data of its subcohort (one major company) where data on primary commuting mode has been collected at the annual health checkup. This company is located in Hitachi city, Ibaraki prefecture, which is a suburban area of East Japan. The type of workplace is a mixture of office and factories. The details of the J-ECOH Study [28] and the present subcohort [29] have been described elsewhere. In Japan, the law requires workers to undergo at least one health examination annually. In this prospective analysis, we included participants who underwent health examinations from April 2006 through March 2011, and the earliest data obtained were considered as baseline (mostly in 2006). Health checkup data obtained 5 year after the baseline examination were collected from April 2011 to March 2016. Prior to the data collection, the conduct of the J-ECOH Study was announced in each participating companies using posters to explain the purpose and procedures of the study. Participants did not provide their verbal or written informed consent to join the study but were allowed to refuse their participation. This consent procedure conforms to the Japanese Ethical Guidelines for Epidemiological Research. The study protocol was approved by the Ethics Committee of the National Center for Global Health and Medicine, Japan.

### Participants

A total of 38,329 participants (32,486 men and 5843 women) aged 30 to 64 years at baseline underwent a baseline health examination and a follow-up examination 5 years after baseline measurement. Of these, 478 participants who had a history of cancer or cardiovascular disease at baseline were excluded because these diseases may influence both commuting mode and BMI. Of the remaining 37,851 participants, we further excluded 7102 (18.6%) who did not have data on commuting mode (*n* = 4326), lifestyle habits (smoking, *n* = 4266; alcohol, *n* = 2783; sleep, *n* = 4272), and occupational factors (overtime work, *n* = 5511; occupational physical activity, *n* = 4444; shift work, *n* = 5225; job position, *n* = 5275) at baseline, leaving 30,749 workers. Some participants met two or more exclusion criteria. Three participants did not have data on body height at baseline, and it was imputed from the following health checkup data. Then, we additionally excluded 991 workers without data on commuting mode after 5-year follow-up, remaining 29,758 participants (25,808 men and 3950 women) aged 30 to 64 years (mean [SD]: 43.2 [8.2] years) for the main analysis. Of these, approximately 0.1 to 3.5% of them did not have 5-year follow-up data of covariates (smoking, *n* = 43; alcohol, *n* = 113; sleep, *n* = 195; leisure-time exercise, *n* = 279; occupational physical activity, *n* = 142; overtime work, *n* = 323; shift work, *n* = 162; job position, *n* = 1040); these missing data of covariates were imputed by using last observation carried forward method. Most of the missing data were replaced by 4-year follow-up data.

### Exposure (primary commuting)

The primary commuting mode to work was self-reported at the annual health examination according to 4 response options of walking, bicycling, train or bus, and car or motorbike. We categorized the 4 types of commuting mode into two groups as (1) inactive commuting (i.e., car or motorbike) and (2) active commuting (i.e., walking or bicycling) or public transportation (train or bus) [20, 30, 31]. We combined active commuting and public transport use as one category because public transport use typically involves in walking [9, 32]. Then, we further reclassified the participants into 4 groups according to the baseline and follow-up commuting mode: (1) maintained inactive commuting, (2) switching from inactive commuting at baseline to active or public transportation mode at 5 years after the baseline examination, (3) switching from active or public transportation mode at baseline to inactive commuting at 5 years after the baseline examination, and (4) maintained active or public transportation mode.

For sensitivity analysis, commuting mode during 1 to 4 years after the baseline period was categorized into the following three categories: persistent inactive commuting, intermittent, and persistent active commuting or public transport use.

### Outcome (obesity indicators)

The body height and weight were measured during annual health examinations, and the BMI of each participant was calculated using the formula kg/m^2^. For the main outcome, we obtained the 5-year changes in BMI by subtracting the baseline BMI from the BMI obtained 5 years after baseline.

Visceral and subcutaneous fat areas and waist circumference were measured in a subgroup of the present participants with additional measurements of these variables (*n* = 4322). Single slice imaging was performed at the umbilical level under fasting condition using a Redix Turbo CT scanner (Hitachi Medico, Tokyo, Japan) and visceral fat area, subcutaneous fat area, and waist circumference were estimated by the PC software application fatPointer (Hitachi Medico, Tokyo, Japan) as described elsewhere [33]. Changes from the baseline examination to 5-year follow-up were also calculated for these three obesity indicators as secondary outcomes.

### Other variables

During examination, the lifestyle habits (smoking, alcohol use, sleep duration, and leisure-time exercise), work-related factors (physical activity at work, overtime work hours, and shift work) and socioeconomic status (job position) of the participants were assessed using a questionnaire. Details of this questionnaire have been explained previously [29]. The metabolic markers for blood pressure, glucose, and lipids were measured following the procedures described elsewhere [34].

### Statistical analysis

We calculated the means (SD) and number (%) to illustrate baseline characteristics of the participants according to the 5-year changes in commuting mode.

Multivariable linear regression was used to quantify the association of 5-year changes in commuting mode with simultaneous BMI changes. We treated maintained inactive commuting mode as reference. First, we adjusted for baseline variables including age (year, continuous), sex, and BMI (kg/m^2^, continuous) as model 1. Then, we adjusted for all factors in model 2, namely, smoking transition (continued no smoking, started smoking, quit smoking, and continued smoking), baseline alcohol use (*go* [unit for Japanese sake; 1 *go* of Japanese sake contains about 23 g of ethanol, which approximates 2 units of alcohol] per day, continuous), 5-year changes in alcohol use (*go*, continuous), baseline sleep duration (< 5 h, 5 to < 6 h, 6 to < 7 h, and ≥ 7 h per day), changes in the sleep duration (decrease, no change, or increase), baseline weekly exercise duration during leisure (min, continuous), 5-year changes in the exercise duration (min, continuous), occupational physical activity transition (continued sedentary work, started sedentary work, started physically active work [standing, walking, or physically active], continued physically active work), job position transition (continued low-rank position, promoted, demoted, and continued high-rank position), shift work transition (continued no shift work, started shift work, quit shift work, and continued shift work), and overtime work transition (continued low overtime work hours, increased overtime work hours, decreased overtime work hours, and continued high overtime work hours). The estimated changes in BMI according to commuting mode were calculated from these models. Subgroup analysis was performed for leisure-time exercise transition (persistently no exercise, quit exercise, started exercise, and persistently active) and occupational physical activity (persistently sedentary work, switched to active work, switched to sedentary work, and persistently active work) in model 2.

We performed the following analyses to confirm the robustness of the results of BMI. First, as about 20% of participants were excluded due to missing data, we performed a sensitivity analysis on the association between commuting and BMI using multiple imputation with chained equations (MICE) for missing data of exposures and covariates at baseline and follow-up. A total of 100 imputed data sets were generated. All analyses were performed on each imputed data set; the 100 estimates were combined into an overall estimate using the rules from Rubin. Second, we conducted an analysis considering the transitions in commuting mode during 1 to 4 years after the baseline. Lastly, we performed an analysis while treating public transport use as an independent category, separated from active commuting.

We repeated the main analysis for other obesity outcomes (visceral fat, subcutaneous fat, and waist circumference) where the baseline obesity outcome was adjusted for in each analysis (e.g., adjustment for baseline visceral fat areas if the outcome is visceral fat change). Two-sided *p*-values of less than 0.05 was considered as statistically significant. All analyses were performed using Stata 14.2 (Stata Corp, College Station, Texas, USA).

## Results

Compared with adults included in the main analysis (n  =  29,758), those excluded due to missing data (n  =  8093) had a higher proportion of women (13.3% vs. 22.5%) and walked less during commuting (< 20 min of walking to and from work, 53.5% vs. 72.6%). They were less likely to engage in long overtime work and sedentary work and be in high job position (Additional file 1: Table S1).

Table 1 shows the characteristics of participants at baseline according to the 5-year changes in commuting mode. Adults who maintained inactive commuting tended to engage in physically active work and shift work compared with the other three groups. They were less likely to be in high position and work less overtime hours. Among adults who maintained inactive commuting and those who switched from inactive commuting, approximately 70% reported < 20 min of walking to and from work at baseline. Data on blood pressure, glycaemic levels and lipid status, which were available for about 80 to 90% of participants, were not substantially different across commuting mode. Baseline walking and cycling during leisure and transitions in leisure-time exercise and occupational physical activity were not largely different across changes in commuting mode (Additional file 1: Table S2).

**Table 1:** Baseline characteristics of the participants according to changes in commuting mode over 5 years (*n* = 29,758)

Baseline variables	Maintained inactive commute^a^ (*n* = 14,704)	Switched from active or public transportation to inactive commute (*n* = 2485)	Switched from inactive to active or public transportation commute (*n* = 2359)	Maintained active or public transportation (*n* = 10,210)
Age, years	43.1 ± 8.1	42.0 ± 8.1	42.1 ± 7.9	43.8 ± 8.2
Men, n (%)	12,405 (84.4)	2147 (86.4)	2023 (85.8)	9233 (90.4)
Body mass index, kg/m^2^	23.5 ± 3.5	23.6 ± 3.5	23.6 ± 3.5	23.6 ± 3.3
Body weight, kg	67.0 ± 12.0	67.9 ± 12.1	67.7 ± 12.2	68.0 ± 11.3
Visceral fat areas, cm^2 b^	119.0 ± 50.7	122.0 ± 48.2	130.6 ± 53.6	119.8 ± 51.3
Subcutaneous fat areas, cm^2 b^	141.2 ± 61.2	146.2 ± 62.0	145.8 ± 59.0	137.8 ± 58.1
Waist circumference, cm^b^	86.4 ± 8.6	87.0 ± 8.4	87.8 ± 8.5	86.3 ± 8.3
Systolic blood pressure, mm Hg	121.7 ± 14.1	121.0 ± 14.2	121.3 ± 14.0	122.1 ± 14.2
Diastolic blood pressure, mm Hg	75.9 ± 10.1	75.5 ± 10.3	75.9 ± 9.9	76.5 ± 10.1
Treatment for hypertension, n (%)	1021 (6.9)	163 (6.6)	135 (5.7)	708 (6.9)
Fasting triglyceride, mg/dL^c^	125.6 ± 89.3	131.8 ± 93.9	131.3 ± 97.0	130.1 ± 95.3
HDL-cholesterol, mg/dL^c^	55.6 ± 14.1	55.5 ± 14.2	55.3 ± 13.9	55.9 ± 14.0
LDL-cholesterol, mg/dL^c^	118.7 ± 30.6	118.1 ± 30.0	118.5 ± 30.6	119.3 ± 29.4
Treatment for dyslipidemia, n (%)	654 (4.5)	96 (3.9)	108 (4.6)	412 (4.0)
Fasting plasma glucose, mg/dL^c^	100.5 ± 18.7	100.5 ± 19.8	99.5 ± 16.5	101.6 ± 19.1
HbA1c, %^c^	5.7 ± 0.7	5.7 ± 0.7	5.6 ± 0.7	5.7 ± 0.7
Treatment for diabetes, n (%)	405 (2.8)	75 (3.0)	50 (2.1)	288 (2.8)
Current smokers, n (%)	7021 (47.7)	3663 (35.9)	1058 (42.6)	1029 (43.6)
Ethanol consumption of ≥46 g per day, n (%)	1291 (8.8)	231 (9.3)	214 (9.1)	977 (9.6)
Sleep duration of < 6 h/day, n (%)	7504 (51.1)	1313 (52.9)	1260 (53.4)	5393 (52.8)
Leisure-time exercise in min/week	56.1 ± 147.6	47.1 ± 107.1	56.8 ± 139.1	48.1 ± 120.6
No leisure-time exercise, n (%)	9567 (65.1)	1663 (66.9)	1513 (64.1)	6838 (67.0)
Sedentary work, n (%)	7810 (53.1)	1660 (66.8)	1588 (67.3)	7486 (73.3)
Shift work, n (%)	3450 (23.5)	372 (15.0)	362 (15.4)	976 (9.6)
Not in high job position, n (%)	12,745 (86.7)	2004 (80.6)	1845 (78.2)	7532 (73.8)
Overtime work ≥45 h/month, n (%)	4987 (33.9)	959 (38.6)	958 (40.6)	3973 (38.9)
Primary commute mode, n (%)				
Walking	0 (0)	659 (26.5)	0 (0)	3017 (29.6)
Cycling	0 (0)	930 (37.4)	0 (0)	1980 (19.4)
Bus/train (public transportation)	0 (0)	896 (36.1)	0 (0)	5213 (51.1)
Car/motorbike	14,704 (100)	0 (0)	2359 (100)	0 (0)
Walking to and from work				
< 20 min, n (%)	10,730 (73.0)	1029 (41.4)	1669 (70.8)	2503 (24.5)
20 to < 40 min, n (%)	3708 (25.2)	969 (39.0)	632 (26.8)	4277 (41.9)
≥ 40 min, n (%)	266 (1.8)	487 (19.6)	58 (2.5)	3430 (33.6)

*HbA1c* hemoglobin A1c, *HDL* high-density lipoprotein, *LDL* low-density lipoprotein

Data are shown as mean ± SD for continuous variables or number (%) for categorical variables

^a^Inactive commuting mode was defined as commuting by car or motorbike

^b^Visceral and subcutaneous fat areas were measured using CT scans among 2129 participants for maintaining inactive commute group, 328 for switching to inactive mode group, 322 for switching to active or public transportation use group, and 1543 for maintaining active or transportation mode group. Meanwhile, the corresponding numbers for CT-scan measured waist circumference were 1672, 256, 266, and 1272, respectively

^c^Data on fasting triglyceride were available among 12,373 participants for maintaining inactive commuting, 2055 for switching to inactive commuting, 1935 for switching to active commuting, and 8753 for maintaining active commuting. For fating or non-fasting HDL, the corresponding numbers were 13,215, 2163, 2052, and 9073; for fasting or non-fasting LDL, 13,118, 2147, 2027, and 9003; for fasting glucose, 12,354, 2050, 1931, and 8742; for HbA1c, 13,195, 2158, 2050, and 9058, respectively

The overall changes in BMI over 5 years were 0.12 kg/m^2^ on average. BMI trajectories over 5 years according to transitions in commuting mode are illustrated in Additional file 1: Figure S1. Table 2 shows the multivariable-adjusted BMI changes according to changes in commuting mode. The maintained inactive commuting group showed some weight gain over the 5-year study period (BMI increase of approximately 0.2 kg/m^2^). Compared with participants who maintained inactive commuting, the participants who switched from active commuting or public transport use to inactive commuting showed higher weight gain. In contrast, the participants who switched from inactive commuting to active commuting or public transport use had lower weight gain. Adults who maintained active commuting or public transportation showed no material weight change. This relationship did not substantially change after adjustment for many potential confounders (model 2); the greatest weight gain was observed in switching to inactive commuting group (0.24 kg/m^2^, 95% CI: 0.20 to 0.27), whereas the least gain in maintained active commuting or public transportation group (0.01 kg/m^2^, 95% CI: − 0.01 to 0.04). A sensitivity analysis using MICE also showed the similar results (Additional file 1: Table S3).

**Table 2:** Changes in body mass index (kg/m^2^) over 5 years as classified according to commuting mode transition (*n* = 29,758)

	Commuting mode transition over 5 years			
	Maintained inactive	Switching to inactive	Switching to active or public transportation	Maintained active or public transportation
Crude model	0.18 (0.16, 0.20)	0.27 (0.21, 0.33)	0.07 (0.01, 0.13)	0.02 (− 0.01, 0.05)
	Reference	*P* = 0.005	P = 0.001	P < 0.001
Multivariable model 1^a^	0.18 (0.16, 0.20)	0.26 (0.23, 0.29)	0.11 (0.09, 0.13)	0.01 (−0.01, 0.04)
	Reference	*P* = 0.006	P < 0.001	*P* < 0.001
Multivariable model 2^b^	0.19 (0.17, 0.21)	0.24 (0.20, 0.27)	0.10 (0.09, 0.12)	0.01 (−0.01, 0.04)
	Reference	P < 0.001	P = 0.001	P < 0.001

Data are shown as estimated mean with 95% confidence intervals. Ref, Reference

^a^Model 1: Baseline variables of age, sex, and body mass index were adjusted

^b^Model 2: Further adjusted for smoking transition, baseline alcohol consumption, 5-year changes in alcohol consumption, baseline sleep duration, changes in the sleep duration, baseline weekly exercise duration during leisure, 5-year changes in the exercise duration, occupational physical activity transition, job position transition, shift work transition, and overtime work transition

When participants were further divided by commuting mode during 1 to 4 years after baseline, maintaining inactive commuting group tended to persistently use inactive commuting during 1 to 4 years after baseline period, and vice versa (Additional file 1: Table S4). The shape of the association between commuting mode and BMI were generally similar to the main results. Participants who engaged in active commuting or public transport use during 1 to 4 years after baseline tended to show less weight gain compared with other groups (Additional file 1: Table S4).

As illustrated in Table 3, subgroup analysis showed a similar tendency in the association of commuting with BMI in subgroups of exercise transitions or occupational physical activity transition. When public transport use was treated as an independent category, the public transport use is beneficially associated with weight gain as with active commuting (Additional file 1: Table S5) even when divided by transitions in exercise or occupational physical activity (Additional file 1: Tables S6 to S7).

**Table 3:** Changes in commuting mode and BMI (kg/m^2^) as classified according to exercise transition or occupational physical activity transition

	Commuting mode transition over 5 years			
	Maintained inactive	Switching to inactive	Switching to active or public transportation	Maintained active or public transportation
Leisure-time exercise				
Maintained no exercise	0.27 (0.24, 0.30)	0.34 (0.30, 0.38)	0.20 (0.18, 0.22)	0.09 (0.06, 0.13)
*n* = 16,087	Reference	*P* < 0.001	*P* = 0.046	*P* < 0.001
Quit exercise	0.34 (0.28, 0.40)	0.41 (0.31, 0.50)	0.25 (0.20, 0.30)	0.18 (0.10, 0.26)
*n* = 6619	Reference	*P* = 0.34	*P* = 0.002	*P* = 0.071
Started exercise	−0.13 (−0.20, −0.07)	−0.12 (− 0.23, − 0.02)	−0.28 (− 0.34, − 0.22)	−0.38 (− 0.46, − 0.29)
*n* = 3494	Reference	*P* = 0.072	*P* = 0.38	*P* = 0.27
Maintained exercise	0.06 (0.02, 0.10)	0.11 (0.04, 0.17)	0.01 (−0.03, 0.04)	−0.07 (− 0.12, − 0.02)
*n* = 6619	Reference	*P* = 0.73	*P* = 0.19	*P* < 0.001
Occupational activity				
Maintained sedentary	0.14 (0.11, 0.16)	0.21 (0.16, 0.25)	0.06 (0.03, 0.08)	−0.03 (−0.06, 0.00)
*n* = 16,985	Reference	P = 0.001	*P* = 0.006	*P* < 0.001
Changed to sedentary	0.43 (0.36, 0.51)	0.45 (0.33, 0.57)	0.41 (0.34, 0.48)	0.35 (0.24, 0.46)
*n* = 1937	Reference	*P* = 0.99	*P* = 0.28	*P* = 0.77
Changed to active	0.00 (−0.08, 0.08)	0.01 (− 0.12, 0.14)	0.03 (− 0.05, 0.12)	−0.09 (− 0.21, 0.03)
*n* = 1559	Reference	*P* = 0.70	*P* = 0.50	*P* = 0.50
Maintained active	0.23 (0.20, 0.26)	0.29 (0.24, 0.35)	0.17 (0.14, 0.20)	0.08 (0.02, 0.13)
*n* = 9277	Reference	*P* = 0.005	*P* = 0.038	*P* = 0.047

Data are shown as estimated mean and 95% confidence intervals. Data were adjusted for factors in model 2

The associations to visceral adiposity (waist circumference, visceral fat areas, and subcutaneous fat areas) among 4322 participants who received visceral adiposity CT scans (maintained inactive group, *n* = 2129; switching from active or public transportation commuting to inactive group, *n* = 328; switching from inactive to active or public transportation commuting group, *n* = 322; maintained active or public transportation commuting group, *n* = 1532) are shown in Table 4. The relationships between commuting mode and the outcomes were less clear than those for BMI. However, maintained inactive commuting tended to show larger gain in these abdominal indicators compared with other commuting patterns (Table 4).

**Table 4:** Changes in indicators of abdominal obesity as classified according to commuting mode transition (*n* = 4322)

	Crude model	*P* values	Multivariable model 1^a^	*P* values	Multivariable model 2^b^	*P* values
Waist circumference (cm)						
Maintained inactive	0.7 (0.4, 1.0)	Reference	0.9 (0.6, 1.1)	Reference	0.9 (0.6, 1.1)	Reference
Switching to inactive	1.2 (0.6, 1.9)	0.20	0.9 (0.5, 1.2)	0.15	0.8 (0.4, 1.2)	0.10
Switching to active or public transportation	−0.4 (−1.1, 0.4)	0.007	0.6 (0.4, 0.8)	0.052	0.5 (0.3, 0.7)	0.17
Maintained active or transportation	0.7 (0.4, 1.1)	0.98	0.7 (0.4, 1.0)	0.85	0.7 (0.4, 1.0)	0.27
Visceral fat areas (cm^2^)						
Maintained inactive	5.3 (3.7, 6.9)	Reference	5.4 (4.1, 6.6)	Reference	5.6 (4.3, 6.9)	Reference
Switching to inactive	6.4 (2.4, 10.3)	0.58	5.2 (3.1, 7.3)	0.63	4.4 (2.3, 6.5)	0.20
Switching to active or public transportation	−0.8 (−5.4, 3.9)	0.007	2.2 (1.1, 3.4)	0.11	1.5 (0.3, 2.7)	0.25
Maintained active or public transportation	4.6 (2.8, 6.4)	0.64	4.2 (2.5, 5.9)	0.42	4.2 (2.5, 5.9)	0.59
Subcutaneous fat areas (cm^2^)						
Maintained inactive	5.1 (3.7, 6.5)	Reference	5.0 (3.9, 6.2)	Reference	5.4 (4.2, 6.5)	Reference
Switching to inactive	5.7 (2.0, 9.5)	0.73	5.5 (3.6, 7.3)	0.55	4.2 (2.4, 6.0)	0.19
Switching to active or public transportation	2.0 (−1.5, 5.5)	0.11	3.2 (1.8, 4.7)	0.16	3.0 (2.0, 4.0)	0.51
Maintained active or public transportation	3.5 (1.9, 5.0)	0.13	3.7 (2.8, 4.7)	0.13	3.2 (1.7, 4.7)	0.80

Data are shown as estimated mean with 95% confidence intervals of changes in obesity outcome. Maintained inactive group, *n* = 2129; switching from active or public transportation commuting to inactive group, *n* = 328; switching from inactive to active or public transportation commuting group, *n* = 322; maintained active or public transportation commuting group, *n* = 1532

^a^Model 1: Adjusted for age and sex at baseline. In addition, baseline variables of each outcome, treated as continuous data, were adjusted for (e.g., baseline waist circumference when outcome is changes in waist circumference)

^b^Model 2: Further adjusted for other variables

## Discussion

In this 5-year observation of approximately 30,000 Japanese workers, maintaining inactive commuting mode was associated with weight gain; compared with this group, switching from active or public transportation commuting to inactive commuting were associated with larger weight gain, and vice versa. Further sensitivity analyses supported the main findings. To the best of our knowledge, this is the first study to estimate the impact of changes in commuting mode to work on body weight and visceral adiposity changes in Asia.

The present analysis showed that adults who maintained inactive commuting gained weight over 5 years (0.19 kg/m^2^), and adults who switched from active or public transportation commuting at baseline to inactive commuting at follow-up had greater weight gain (0.24 kg/m^2^) than those who maintained inactive commuting. By contrast, those who switched to and those who maintained active commuting or public transportation showed less weight gain than those who maintaining inactive commuting (0.10 kg/m^2^ and 0.01 kg/m^2^, respectively). Prospective studies [19–21, 23] have also shown similar trends of changes in BMI according to changes in commuting mode, although categories of the changes in exposure is somewhat heterogeneous. Additionally, an Australian study reported that persistent active commuting was associated with a lower BMI [22]. A recent systematic review also showed that public transport use is associated with a lower BMI [35]. Collectively, these findings indicate that switching from inactive commuting mode to more physically active commuting and maintaining active commuting or public transport use over time may be beneficially associated with body weight among working adults.

In the present data, adults who switched to inactive commuting tended to show greater weight gain compared with other groups. A UK study also reported a similar finding [21]. The reason of the greater gain is unclear. However, in theory, reductions in energy expenditure may lead to difficulty in maintain energy balance [36], resulting in weight gain [36]. As the related data are sparse, additional studies on this issue, as well as studies which can elucidate the underlying mechanisms are warranted.

The extent of BMI change in the present study was smaller than those in the comparable longitudinal studies [19–21, 23]. This is may be due to that average BMI level in the present study is lower than those in the previous Western studies [19–21, 23] (23.5 kg/m^2^ vs. approximately 24 to 27 kg/m^2^); the extent of weight change in Asians may be smaller than Caucasians. Another reason of smaller changes in BMI in the present study might be partly explained by the differences in the assessment of BMI. The present study used objectively measured body height and weight, whereas most of the previous studies used self-report [19, 21, 23]. Nonetheless, Asians tend to develop diabetes even with a small weight gain [37] and accumulating evidence suggest that obesity even without metabolic abnormality may increase risk of all-cause mortality and cardiovascular disease [38]. Although weight change associated with commuting mode was small, the present results would provide important viewpoint to prevent or delay the onset of non-communicable diseases in Asians. Besides obesity, promotion of active commuting and public transport use would help solve health-related problems including traffic-related air pollution and noise [11]. Given the limited available data, further large studies from Asia are needed to establish the solid evidence on commuting and BMI in Asia.

Our novel finding is that the beneficial association of active or public transportation commuting with BMI was observed across different transitions of leisure-time exercise and occupational physical activity, while both leisure-time exercise and occupational physical activity were inversely associated with BMI over time. Similarly with the present findings, observational studies have shown that exercise was associated with weight reduction [39]. Although prospective data are sparse, increase in occupational physical activity over time was inversely associated with BMI [20, 40]. Importantly, the present data showed that maintaining or switching to active commuting or public transport use are respectively associated with less weight gain even among those who did not engage in exercise and those who engaged in sedentary at work (Additional file 1: S6 and S7). Both active commuting and public transport use may be a good source of physical activity among adults who are inactive during leisure or at workplace.

In the present analysis for abdominal obesity (i.e., waist circumference, visceral fat areas, and subcutaneous fat areas) measured by CT scans, maintaining inactive commuting tended to show larger gain in these parameters than other commuting patterns. However, these changes did not reach significance and were somewhat different from those of BMI. The non-significant and less clear changes may be due to the small sample size with abdominal CT measurements in our data set when participants were divided according to commuting mode transition (especially for those who changed the commuting mode, *n* = 320 to 330). Nonetheless, recent randomised controlled trials showed that active commuting by cycling reduced body fat mass compared with no intervention [41, 42]. Although we are not aware of any observational study on the prospective association of active commuting to work with visceral adiposity, one cross-sectional study in the UK [30] reported that active commuters tended to show lower visceral adipose tissue mass compared with those commuting only by car. By contrast, another cross-sectional study in Thailand [43] reported an opposite finding and found higher odds of having central obesity in adults who were actively commuting; their finding may be ascribed to reverse causality. As available longitudinal data are still limited, further large-scale studies are needed to elucidate the association of active commuting and public transport use with abdominal obesity.

The present data showed that leisure-time exercise and occupational physical activity were not largely different across different transitions in commuting mode; only exception was that adults maintaining inactive commuting were less likely to engage in sedentary work. These results are somewhat different from recent findings from a cross-sectional study [44] showing that walking during commuting was positively associated with cycling during leisure and negatively associated with leisure-time physical activity among French adults. Although the causal-relationship among domain-specific physical activities is unclear, this kind of information would help detect high risk population of physical inactivity, which should be a target for physical activity intervention.

It would be important to discuss implication of the present findings for physical activity promotion. In Japan, Ministry of Land, Infrastructure, Transport and Tourism has engaged in policies on promoting active commuting including walking and cycling and switching from car commuting to public transport use [45]. In addition, Japan Sport Agency, which is under Ministry of Education, Culture, Sports, Science and Technology, recently launched a project for promoting walking during commuting or at workplace especially among working adults [46]. However, supporting evidence for these actions, especially for switching to public transport use, has been lacking in relation to health. Therefore, the present data would support high level policy makers and other stakeholders (e.g., local governments, transport agencies, health professionals) to advance such actions from the viewpoint of health promotion. As summarized by Sallis et al. [47] and declared by Japanese Association of Exercise Epidemiology [48] recently, providing scientific knowledge including the present findings to such stakeholders would be important to ensure evidence-based policy making in Japan.

The strengths of this study include its longitudinal design that provides more convincing evidence than cross-sectional design. Further, this is the largest study on this topic to date, enabling us to detect an association with a greater statistical power. We used objectively measured BMI and visceral adiposity.

The limitations of this study also need to be clarified. First, we assessed the primary commuting mode via self-reports. Therefore, random misclassifications might have diluted the results. Second, the present questionnaire for commuting mode was not designed to estimate the precise volume or intensity of physical activity and sedentary behaviour during commuting, which may have an impact on body weight and composition [49–52]. Although we are not aware of any Asian studies showing time spent in walking during commuting by commuting mode, our findings are similar to those from a recent UK study [53] reporting that approximately 20 to 30 min of walking per day accumulated during public transport. It is possible that public transport users with shorter time spent in walking and/or cycling to public transport access points may benefit less than those with longer time [30]. In addition, we found that, among participants who persistently used public transport as main commuting mode, longer time spent in walking to and from work was related to less weight gain (Additional file 1: Table S8). Third, unmeasured and/or residual confounders may have affected the results, although we adjusted for many covariates. Fourth, we do not have data on the reason for the changes in commuting mode. Some participants might have switched to active commuting to control body weight or health condition and such people may tend to adhere to multiple healthy lifestyles. However, baseline levels of BMI and other metabolic markers including blood pressures, glucose, and lipids were not substantially different across the various transitions of commuting mode and we adjusted for multiple lifestyle factors. Therefore, such possibility might be low. Fifth, approximately 20% of participants were excluded from the main analysis due to missing data, and some characteristics were slightly different between those included and excluded. However, our sensitivity analysis using MICE showed similar results with those from the primary analysis. Sixth, timing of switching in commuting mode would affect the results as the exposure period is different. However, we did not further examine this issue in detail as sample size is insufficient when participants were further divided by the timing of the change, although we roughly considered the transitions in commuting mode during 1 to 4 years after baseline in an additional analysis. Future studies using different definition of switching (e.g., participant reported car use in the first year, but switched to walking in the second year, then examine the change in the outcomes between the first and third year) are needed. Seventh, we examined the association of 5-year changes in exposure with simultaneous changes in outcomes. Another analytical approach including difference-in-difference analysis with considering non-linear association [54, 55] would help understand the longitudinal relationship between commuting mode and health outcomes. Lastly, the data were obtained from workers in a large-scale company in Japan and the majority were men. Therefore, caution should be exercised when generalising the present findings to workers in small to medium-sized enterprises and women.

In conclusion, we found that switching from inactive commuting to active or public transportation commuting was associated with less BMI increase. Meanwhile, switching from active commuting or public transport use to inactive commuting was associated with higher increase in BMI among Japanese men and women. This relationship was mostly unchanged across different transitions of leisure-time exercise or occupational physical activity. The promotion of active commuting and/or public transportation use at the population level by various stakeholders, including policy makers, local government officials, and companies, would help to end the global obesity epidemic.

## Supplementary information

**Additional file 1: Table S1.** Characteristics of participants included and excluded from the main analysis. **Table S2.** Association of changes in commuting mode with baseline exercise and transitions in leisure-time exercise or occupational physical activity. **Table S3.** A sensitivity analysis on the changes in commuting mode and BMI with multiple imputation using chained equations (*n* = 37,847). **Table S4.** A sensitivity analysis for BMI with consideration of transitions in commuting mode during 1 to 4 years after baseline (*n* = 24,686). **Table S5.** Associations of active commuting, public transport use, and inactive commuting with BMI. **Table S6.** Associations of active commuting, public transport use, and inactive commuting with BMI by leisure-time exercise transition. **Table S7.** Associations of active commuting, public transport use, and inactive commuting with BMI by occupational physical activity transition. **Table S8.** Changes in BMI over 5 years according to the transitions in time spent in walking to and from work among workers who persistently used public transport as main commuting mode **Figure S1.** Trajectories of non-adjusted average body mass index according to the changes in commuting mode.

## Data Availability

The datasets generated and/or analysed during the current study are not publicly available due ethical restrictions and participant confidentiality concerns, but de-identified data are available from Dr. Mizoue (Department of Epidemiology and Prevention, Center for Clinical Sciences, National Center for Global Health and Medicine, Tokyo, Japan) to qualified researchers on reasonable request.

## Abbreviations

BMI: Body mass index
CI: Confidence interval
CT: Computed tomography
J-ECOH: Japan Epidemiology Collaboration on Occupational Health
MICE: Multiple imputation with chained equations
