# Supplementary material for: Association of changes in commute mode with body mass index and visceral adiposity: a longitudinal study
Source: Int J Behav Nutr Phys Act. 2019 Nov 6;16:101. doi: 10.1186/s12966-019-0870-x (PMC6836489; doi:10.1186/s12966-019-0870-x)
Supplement: Supplementary file 1 — Additional file 1: Table S1. Characteristics of participants included and excluded from the main analysis. Table S2. Association of changes in commuting mode with baseline exercise and transitions in leisure-time exercise or occupational physical activity. Table S3. A sensitivity analysis on the changes in commuting mode and BMI with multiple imputation using chained equations (n = 37,847). Table S4. A sensitivity analysis for BMI with consideration of transitions in commuting mode during 1 to 4 years after baseline (n = 24,686). Table S5. Associations of active commuting, public transport use, and inactive commuting with BMI. Table S6. Associations of active commuting, public transport use, and inactive commuting with BMI by leisure-time exercise transition. Table S7. Associations of active commuting, public transport use, and inactive commuting with BMI by occupational physical activity transition. Table S8. Changes in BMI over 5 years according to the transitions in time spent in walking to and from work among workers who persistently used public transport as main commuting mode Figure S1. Trajectories of non-adjusted average body mass index according to the changes in commuting mode. [file 12966_2019_870_MOESM1_ESM.pdf]

Supplementary data

Association of changes in commute mode with body mass index and visceral adiposity

Kuwahara K,[1,2,*] Noma H,[3] Nakagawa T,[4] Honda T,[4] Yamamoto S,[4] Hayashi T,[4] Mizoue T.[1]

Table of contents: **Page no.**

Supplementary Table 1……………………………………………………S2
Supplementary Table 2……………………………………………………S3
Supplementary Table 3……………………………………………………S4
Supplementary Table 4……………………………………………………S5-S6
Supplementary Table 5……………………………………………………S7-S8
Supplementary Table 6……………………………………………………S9
Supplementary Table 7……………………………………………………S10
Supplementary Table 8……………………………………………………S11
Supplementary Figure 1…………………………………………………..,S12

Supplementary Table 1. Characteristics of participants included and excluded from the main analysis

Baseline variables	Included (n=29,758)	Exclude (n=8093)
Age, year	43.2 (8.2)	42.2 (8.7)
Female	13.3	22.5
Commuting mode		
Walking	12.4	11.1
Cycling	9.8	9.3
Public transport	20.5	19.7
Car/motorbike	57.3	59.9
<20 min of walking to and from work	53.5	72.6
Shift work	17.3	19.9
≥45 hours of overtime work	36.5	28.6
Sedentary work	62.3	46.9
High job position	18.9	11.7
Current smoking	42.9	38.2
Heavy alcohol use*	9.1	5.0
No exercise	65.8	70.4
Short sleep duration (<5 hrs/day)	7.3	8.5
BMI, kg/m^2	23.5 (3.4)	23.3 (3.5)
SBP, mmHg	122 (14)	121 (15)
DBP, mmHg	76 (10)	75 (11)
Treatment of hypertension	6.8	5.0
Fasting triglycerides, mg/dL	128 (92)	119 (94)
HDL-cholesterol, mg/dL	56 (14)	58 (15)
LDL-cholesterol, mg/dL	119 (30)	118 (31)
Treatment of dyslipidemia	4.3	2.7
Fasting plasma glucose, mg/dL	101 (19)	98 (19)
HbA1c, %	5.7 (0.7)	5.7 (0.8)
Treatment of diabetes	2.8	2.2

Data are shown as % or mean (SD). BMI, body mass index; DBP, diastolic blood pressure; HDL, high-density lipoprotein; LDL, low-density lipoprotein; SBP, systolic blood pressure.

* Heavy alcohol use was defined as consumption of ≥2 *go* of Japanese sake equivalent per day, which approximates to 46 g or more of ethanol consumption per day.

Supplementary Table 2. Association of changes in commuting mode with baseline exercise and transitions in leisure-time exercise or occupational physical activity

	Maintained inactive commute (n=14,704)	Switched from active or public transportation to inactive commute (n=2485)	Switched from inactive to active or public transportation commute (n=2359)	Maintained active or public transportation (n=10,210)
Baseline leisure-time exercise				
Some walking, n (%)	1626 (11.1)	229 (9.2)	263 (11.2)	1091 (10.7)
Some cycling (10 km/ occasion), n (%)	211 (1.4)	60 (2.4)	40 (1.7)	268 (2.6)
Transitions in leisure-time exercise				
Maintained no exercise	7855 (53.4)	1330 (53.5)	1241 (52.6)	5661 (55.5)
Quit exercise	1803 (12.3)	305 (12.3)	295 (12.5)	1155 (11.3)
Started exercise	1712 (11.6)	333 (13.4)	272 (11.5)	1177 (11.5)
Maintained exercise	3334 (22.7)	517 (20.8)	551 (23.4)	2217 (21.7)
Transitions in occupational physical activity				
Maintained sedentary	6989 (47.5)	1481 (59.6)	1472 (62.4)	7043 (69.0)
Changed to sedentary	1035 (7.0)	160 (6.4)	175 (7.4)	567 (5.6)
Changed to active	821 (5.6)	179 (7.2)	116 (4.9)	443 (4.3)
Maintained active	5859 (39.9)	665 (26.8)	596 (25.3)	2157 (21.1)

Data are shown as n (%).

Supplementary Table 3. A sensitivity analysis on the changes in commuting mode and BMI with multiple imputation using chained equations (n=37,847)

	Multivariable model 2	P values
Maintained inactive	0.17 (0.15, 0.19)	Reference
Switching to inactive	0.24 (0.19, 0.29)	0.018
Switching to active or public transportation	0.07 (0.01, 0.13)	<0.001
Maintained active or public transportation	0.04 (0.01, 0.06)	<0.001

Data are shown as adjusted mean with 95% confidence intervals in body mass index (kg/m^2) over 5 years.

Supplementary Table 4. A sensitivity analysis for BMI with consideration of transitions in commuting mode during 1 to 4 years after baseline (n = 24,686)

Commuting mode	N	Non-adjusted	Multivariable-adjusted 1*	Multivariable-adjusted 2†
Persistently inactive				
Inactive during 1 to 4 yrs after baseline	10,306 (83.7)	0.16 (0.13, 0.19)	0.18 (0.15, 0.20)	0.18 (0.16, 0.21)
Intermittent during 1 to 4 yrs after baseline	1,968 (16.0)	0.20 (0.13, 0.26)	0.17 (0.15, 0.19)	0.17 (0.15, 0.19)
Active or public transport use during 1 to 4 yrs after baseline	34 (0.3)	0.06 (-0.45, 0.57)	0.01 (-0.01, 0.03)	0.03 (0.00, 0.05)
Became inactive				
Inactive during 1 to 4 yrs after baseline	656 (33.1)	0.29 (0.18, 0.40)	0.23 (0.20, 0.26)	0.22 (0.19, 0.25)
Intermittent during 1 to 4 yrs after baseline	1,037 (52.3)	0.29 (0.20, 0.39)	0.25 (0.22, 0.28)	0.22 (0.19, 0.25)
Active or public transport use during 1 to 4 yrs after baseline	289 (14.6)	0.21 (0.02, 0.40)	0.21 (0.19, 0.24)	0.18 (0.15, 0.21)
Became active				
Inactive during 1 to 4 yrs after baseline	545 (29.1)	0.07 (-0.05, 0.19)	0.11 (0.10, 0.13)	0.11 (0.09, 0.13)
Intermittent during 1 to 4 yrs after baseline	1,038 (55.3)	0.06 (-0.03, 0.15)	0.07 (0.05, 0.09)	0.08 (0.05, 0.09)
Active or public transport use during 1 to 4 yrs after baseline	293 (15.6)	0.16 (-0.01, 0.32)	0.11 (0.10, 0.13)	0.09 (0.07, 0.11)
Persistently active				
Inactive during 1 to 4 yrs after baseline	89 (1.0)	0.05 (-0.26, 0.37)	0.02 (0.00, 0.05)	0.05 (0.02, 0.08)
Intermittent during 1 to 4 yrs after baseline	625 (7.3)	0.13 (0.01, 0.24)	0.03 (0.01, 0.05)	0.03 (0.01, 0.05)
Active or public transport use during 1 to 4 yrs after baseline	7,806 (91.6)	0.06 (-0.04, 0.03)	0.00 (-0.03, 0.03)	-0.01 (-0.04, 0.02)

Data are shown as changes in BMI over 5 years with 95% confidence intervals.

*Adjusted for baseline variables of age, sex, and body mass index.
†Further adjusted for smoking transition, baseline alcohol consumption, 5-year changes in alcohol consumption, baseline sleep duration, changes in the sleep duration, baseline weekly exercise duration during leisure, 5-year changes in the exercise duration, occupational physical activity transition, job position transition, shift work transition, and overtime work transition.

Supplementary Table 5. Associations of active commuting, public transport use, and inactive commuting with BMI.

	Crude model	P values	Multivariable model 1*	P values	Multivariable model 2†	P values
Maintained inactive (n = 14,704)	0.18 (0.16, 0.20)	Ref	0.18 (0.16, 0.20)	Ref	0.19 (0.17, 0.21)	Ref
Switching from transport to inactive (n = 896)	0.29 (0.20, 0.39)	0.023	0.24 (0.21, 0.27)	0.007	0.21 (0.18, 0.24)	<0.001
Switching from active to inactive (n = 1589)	0.26 (0.18, 0.33)	0.049	0.24 (0.21, 0.26)	0.12	0.22 (0.20, 0.25)	0.026
Maintained transport (n = 4580)	0.01 (-0.03, 0.05)	<0.001	0.02 (0.00, 0.04)	<0.001	0.02 (0.00, 0.04)	<0.001
Switching from inactive to transport (n = 910)	0.07 (-0.02, 0.17)	0.029	0.11 (0.09, 0.13)	0.11	0.10 (0.08, 0.12)	0.13
Switching from active to transport (n = 932)	0.07 (-0.03, 0.16)	0.02	0.12 (0.10, 0.13)	0.008	0.10 (0.08, 0.12)	0.099
Maintained active (n = 4065)	0.04 (-0.01, 0.08)	<0.001	0.03 (0.01, 0.06)	<0.001	0.03 (0.01, 0.06)	<0.001
Switching from inactive to active (n = 1449)	0.07 (0.00, 0.15)	0.006	0.01 (-0.02, 0.05)	0.001	0.02 (-0.01, 0.06)	0.001
Switching from transport to active (n = 633)	-0.09 (-0.21, 0.02)	<0.001	-0.09 (-0.13, -0.05)	<0.001	-0.09 (-0.13, -0.05)	0.002

Data are shown as estimated mean with 95% confidence intervals. Ref, Reference.

*Model 1: Baseline variables of age, sex, and body mass index were adjusted.

†Model 2: Further adjusted for smoking transition, baseline alcohol consumption, 5-year changes in alcohol consumption, baseline sleep duration,

changes in the sleep duration, baseline weekly exercise duration during leisure, 5-year changes in the exercise duration, occupational physical activity transition, job position transition, shift work transition, and overtime work transition.

Supplementary Table 6. Associations of active commuting, public transport use, and inactive commuting with BMI by leisure-time exercise transition

	Leisure-time exercise transition			
Commuting mode transition	Maintained no exercise	Quit exercise	Started exercise	Maintained exercise
Maintained inactive	0.27 (0.24, 0.30)	0.35 (0.29, 0.40)	-0.12 (-0.19, -0.06)	0.06 (0.02, 0.09)
	Reference	Reference	Reference	Reference
Switching from transport to inactive	0.32 (0.27, 0.36)	0.42 (0.33, 0.52)	-0.17 (-0.28, -0.06)	0.07 (0.00, 0.13)
	P=0.001	P=0.85	P=0.20	P=0.15
Switching from active to inactive	0.32 (0.28, 0.35)	0.39 (0.31, 0.46)	-0.09 (-0.18, -0.01)	0.10 (0.05, 0.14)
	P=0.013	P=0.29	P=0.16	P=0.16
Maintained transport	0.10 (0.07, 0.13)	0.16 (0.10, 0.23)	-0.36 (-0.43, -0.29)	-0.06 (-0.10, -0.02)
	P=0.004	P=0.14	P=0.65	P=0.004
Switching from inactive to transport	0.19 (0.17, 0.22)	0.22 (0.17, 0.28)	-0.26 (-0.32, -0.20)	0.01 (-0.03, 0.04)
	P=0.39	P=0.06	P=0.59	P=0.12
Switching from active to transport	0.19 (0.16, 0.21)	0.26 (0.20, 0.32)	-0.34 (-0.41, -0.27)	0.00 (-0.03, 0.04)
	P=0.16	P=0.95	P=0.31	P=0.31
Maintained active	0.12 (0.08, 0.15)	0.21 (0.12, 0.29)	-0.37 (-0.46, -0.28)	-0.04 (-0.09, 0.01)
	P<0.001	P=0.095	P=0.002	P=0.002
Switching from inactive to active	0.12 (0.07, 0.16)	0.17 (0.07, 0.27)	-0.38 (-0.50, -0.27)	-0.06 (-0.13, 0.00)
	P=0.051	P=0.011	P=0.13	P=0.61
Switching from transport to active	0.00 (-0.05, 0.06)	0.10 (-0.02, 0.22)	-0.52 (-0.66, -0.39)	-0.17 (-0.25, -0.09)
	P=0.083	P=0.79	P=0.13	P=0.13

Data are adjusted for factors in model 2.

Supplementary Table 7. Associations of active commuting, public transport use, and inactive commuting with BMI by occupational physical activity transition

	Occupational physical activity transition			
	Maintained sedentary (n=16,985)	Changed to sedentary (n=1937)	Changed to active (n=1559)	Maintained active (n=9277)
Maintained inactive	0.14 (0.11, 0.17)<br>Reference	0.43 (0.35, 0.52)<br>Reference	0.00 (-0.09, 0.09)<br>Reference	0.23 (0.20, 0.27)<br>Reference
Switching from transport to inactive	0.20 (0.16, 0.24)<br>P=0.018	0.36 (0.28, 0.44)<br>P=0.14	-0.08 (-0.17, 0.00)<br>P=0.62	0.20 (0.17, 0.23)<br>P=0.012
Switching from active to inactive	0.18 (0.14, 0.21)<br>P=0.014	0.45 (0.38, 0.52)<br>P=0.30	0.02 (-0.06, -0.10)<br>P=0.38	0.21 (0.19, 0.24)<br>P=0.073
Maintained transport	-0.01 (-0.04, 0.01)<br>P<0.001	0.34 (0.25, 0.44)<br>P=0.77	-0.14 (-0.25, -0.03)<br>P=0.36	0.09 (0.04, 0.13)<br>P=0.23
Switching from inactive to transport	0.06 (0.04, 0.08)<br>P=0.30	0.40 (0.32, 0.47)<br>P=0.17	0.07 (-0.02, 0.15)<br>P=0.37	0.17 (0.13, 0.20)<br>P=0.27
Switching from active to transport	0.08 (0.06, 0.10)<br>P=0.16	0.38 (0.29, 0.47)<br>P=0.11	-0.01 (-0.10, 0.09)<br>P=0.53	0.12 (0.08, 0.15)<br>P=0.94
Maintained active	-0.03 (-0.06, 0.02)<br>P<0.001	0.40 (0.29, 0.51)<br>P=0.55	-0.03 (-0.16, 0.09)<br>P=0.63	0.12 (0.07, 0.17)<br>P=0.13
Switching from inactive to active	-0.05 (-0.09, 0.00)<br>P=0.003	0.40 (0.27, 0.53)<br>P=0.72	0.00 (-0.15, 0.15)<br>P=0.85	0.12 (0.05, 0.18)<br>P=0.073
Switching from transport to active	-0.14 (-0.19, -0.09)<br>P=0.003	0.24 (0.09, 0.40)<br>P=0.32	-0.15 (-0.32, 0.03)<br>P=0.16	0.03 (-0.04, 0.10)<br>P=0.08

Data were adjusted for factors in model 2.

Supplementary Table 8. Changes in BMI over 5 years according to the transitions in time spent in walking to and from work among workers who persistently used public transport as main commuting mode

Time spent in walking to and from work	Crude model	Multivariable model 1*	Multivariable model 2†
Persistently short‡ (n=204)	0.08 (-0.13, 0.28)	0.05 (0.02, 0.07)	0.05 (0.03, 0.08)
Intermediate (n=3215)	0.01 (-0.04, 0.06)	0.02 (0.00, 0.04)	0.02 (0.00, 0.04)
Persistently long‡(n=1161)	0.01 (-0.07, 0.09)	0.02 (0.00, 0.04)	0.01 (-0.01, 0.04)

Data are shown as estimated mean with 95% confidence intervals.

*Model 1: Baseline variables of age, sex, and body mass index were adjusted.

†Model 2: Further adjusted for smoking transition, baseline alcohol consumption, 5-year changes in alcohol consumption, baseline sleep duration, changes in the sleep duration, baseline weekly exercise duration during leisure, 5-year changes in the exercise duration, occupational physical activity transition, job position transition, shift work transition, and overtime work transition.

‡ Persistently short is defined as <20 min of walking to and from walk both at baseline and 5 year follow-up examination, whereas persistently long was defined as 40 min or more of walking to and from work both at baseline and 5 year follow-up examination.

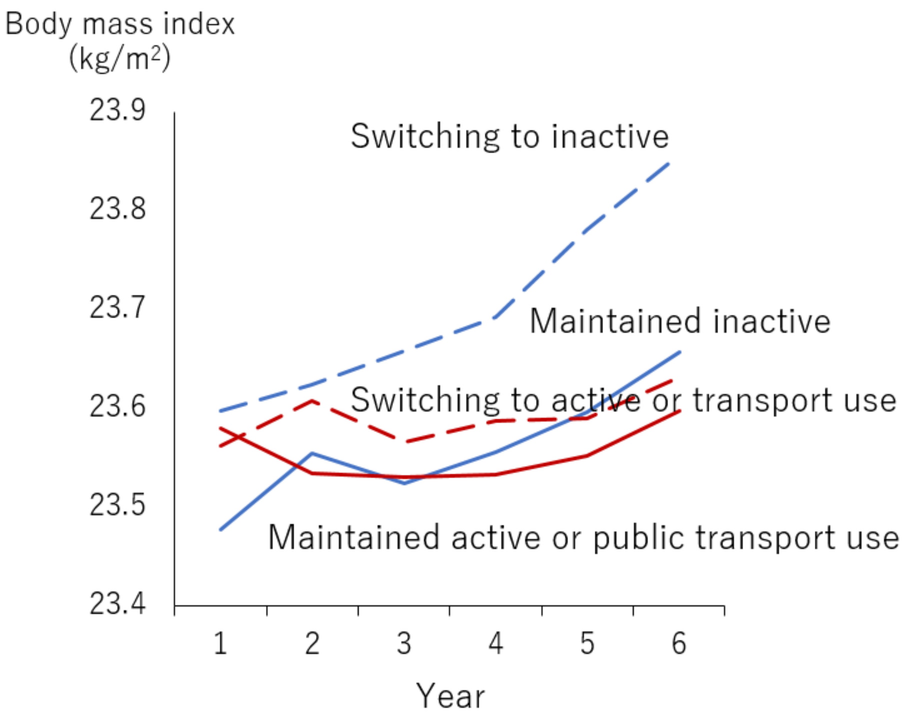

Supplementary Figure 1. Trajectories of non-adjusted average body mass index according to the changes in commuting mode.

Dashed line in blue indicates “Switching to inactive group”, straight line in blue indicates “Maintained inactive group”, dashed line in red indicates “Switching to active or transport use group”, and straight line in red indicates “Maintained active or public transport use group”. BMI trajectories from baseline (Year 1) to 5-year follow-up (Year 6) were calculated among 29 758 participants; 393 were excluded due to no data on BMI during 1 year to 4 years after the baseline examination (Year 2 to 5).
